# Supplementary material for: Role and mechanism of NCAPD3 in promoting malignant behaviors in gastric cancer
Source: Front Pharmacol. 2024 Apr 22;15:1341039. doi: 10.3389/fphar.2024.1341039 (PMC11070777; doi:10.3389/fphar.2024.1341039)
Supplement: Supplementary file 11 [file DataSheet2.ZIP › GSEA/GO_CC/my_analysis.Gsea.1599461408869/GO_GOLGI_MEMBRANE.html]

Details for gene set GO\_GOLGI\_MEMBRANE[GSEA]

|  || Dataset | filtered\_dataset.sample\_info.cls#WT\_versus\_NCAPD3\_MUT |
| Phenotype | sample\_info.cls#WT\_versus\_NCAPD3\_MUT |
| Upregulated in class | NCAPD3\_MUT |
| GeneSet | GO\_GOLGI\_MEMBRANE |
| Enrichment Score (ES) | -0.24290282 |
| Normalized Enrichment Score (NES) | -1.5521322 |
| Nominal p-value | 0.04812834 |
| FDR q-value | 0.120855026 |
| FWER p-Value | 0.842 |
Table: GSEA Results Summary

  

Fig 1: Enrichment plot: GO\_GOLGI\_MEMBRANE      
 Profile of the Running ES Score & Positions of GeneSet Members on the Rank Ordered List

  

| SYMBOL | TITLE | RANK IN GENE LIST | RANK METRIC SCORE | RUNNING ES | CORE ENRICHMENT || 1 | 222236 | NAPEPLD | 36 | 0.977 | 0.0078 | No |
| 2 | 55343 | SLC35C1 | 92 | 0.804 | -0.0045 | No |
| 3 | 51643 | TMBIM4 | 129 | 0.773 | -0.0038 | No |
| 4 | 8549 | LGR5 | 148 | 0.739 | 0.0089 | No |
| 5 | 29940 | DSE | 174 | 0.703 | 0.0151 | No |
| 6 | 126792 | B3GALT6 | 223 | 0.658 | 0.0029 | No |
| 7 | 55275 | VPS53 | 278 | 0.609 | -0.0154 | No |
| 8 | 25972 | UNC50 | 297 | 0.593 | -0.0078 | No |
| 9 | 2524 | FUT2 | 312 | 0.587 | 0.0024 | No |
| 10 | 55697 | VAC14 | 329 | 0.576 | 0.0109 | No |
| 11 | 55676 | SLC30A6 | 353 | 0.565 | 0.0138 | No |
| 12 | 23315 | SLC9A8 | 398 | 0.537 | 0.0002 | No |
| 13 | 9743 | ARHGAP32 | 413 | 0.528 | 0.0085 | No |
| 14 | 56894 | AGPAT3 | 414 | 0.527 | 0.0269 | No |
| 15 | 4247 | MGAT2 | 432 | 0.513 | 0.0324 | No |
| 16 | 22796 | COG2 | 445 | 0.507 | 0.0414 | No |
| 17 | 29071 | C1GALT1C1 | 457 | 0.500 | 0.0508 | No |
| 18 | 7109 | TRAPPC10 | 475 | 0.488 | 0.0554 | No |
| 19 | 9653 | HS2ST1 | 591 | 0.429 | -0.0140 | No |
| 20 | 113189 | CHST14 | 622 | 0.411 | -0.0217 | No |
| 21 | 11226 | GALNT6 | 659 | 0.397 | -0.0342 | No |
| 22 | 51000 | SLC35B3 | 740 | 0.355 | -0.0805 | No |
| 23 | 256435 | ST6GALNAC3 | 766 | 0.341 | -0.0870 | No |
| 24 | 1718 | DHCR24 | 832 | 0.293 | -0.1244 | No |
| 25 | 7357 | UGCG | 852 | -0.254 | -0.1295 | No |
| 26 | 50640 | PNPLA8 | 881 | -0.296 | -0.1397 | No |
| 27 | 51124 | IER3IP1 | 918 | -0.347 | -0.1540 | No |
| 28 | 966 | CD59 | 1040 | -0.436 | -0.2276 | Yes |
| 29 | 7132 | TNFRSF1A | 1055 | -0.449 | -0.2222 | Yes |
| 30 | 2523 | FUT1 | 1068 | -0.456 | -0.2150 | Yes |
| 31 | 339122 | RAB43 | 1083 | -0.462 | -0.2091 | Yes |
| 32 | 1604 | CD55 | 1090 | -0.468 | -0.1971 | Yes |
| 33 | 857 | CAV1 | 1098 | -0.475 | -0.1856 | Yes |
| 34 | 3340 | NDST1 | 1104 | -0.481 | -0.1724 | Yes |
| 35 | 3134 | HLA-F | 1139 | -0.506 | -0.1796 | Yes |
| 36 | 9245 | GCNT3 | 1147 | -0.511 | -0.1669 | Yes |
| 37 | 56180 | MOSPD1 | 1186 | -0.558 | -0.1752 | Yes |
| 38 | 58480 | RHOU | 1189 | -0.560 | -0.1571 | Yes |
| 39 | 5154 | PDGFA | 1191 | -0.566 | -0.1380 | Yes |
| 40 | 23461 | ABCA5 | 1206 | -0.583 | -0.1278 | Yes |
| 41 | 83548 | COG3 | 1211 | -0.588 | -0.1102 | Yes |
| 42 | 2734 | GLG1 | 1240 | -0.618 | -0.1091 | Yes |
| 43 | 10802 | SEC24A | 1251 | -0.639 | -0.0940 | Yes |
| 44 | 63971 | KIF13A | 1256 | -0.645 | -0.0744 | Yes |
| 45 | 1956 | EGFR | 1266 | -0.657 | -0.0580 | Yes |
| 46 | 55638 | SYBU | 1275 | -0.663 | -0.0406 | Yes |
| 47 | 2650 | GCNT1 | 1342 | -0.781 | -0.0617 | Yes |
| 48 | 23431 | AP4E1 | 1359 | -0.827 | -0.0445 | Yes |
| 49 | 8548 | BLZF1 | 1398 | -1.103 | -0.0338 | Yes |
| 50 | 2591 | GALNT3 | 1403 | -1.195 | 0.0051 | Yes |
Table: GSEA details [plain text format]

  

Fig 2: GO\_GOLGI\_MEMBRANE      
 Blue-Pink O' Gram in the Space of the Analyzed GeneSet

  

Fig 3: GO\_GOLGI\_MEMBRANE: Random ES distribution      
 Gene set null distribution of ES for **GO\_GOLGI\_MEMBRANE**
